# Green synthesized gold nanoparticles and CuO-based nonenzymatic sensor for saliva glucose monitoring†

**DOI:** 10.1039/d3ra05644a

**Published:** 2024-01-02

**Authors:** Md Younus Ali, Heman B. Abdulrahman, Wei-Ting Ting, Matiar M. R. Howlader

**Affiliations:** a Department of Electrical and Computer Engineering, McMaster University 1280 Main Street West Hamilton ON L8S 4K1 Canada howladm@mcmaster.ca

## Abstract

Glucose, essential for brain and muscle functions, requires careful monitoring in diabetes and other chronic disease management. While blood glucose monitoring provides precise information about these diseases, it remains an invasive method. Saliva glucose monitoring could offer an alternative approach, but the glucose concentration in saliva is very low. In this work, we report a simple, low-cost, highly sensitive nonenzymatic electrochemical glucose sensor. We developed this sensor using green synthesized gold nanoparticles (AuNPs) and wet chemical synthesized copper oxide (CuO) nanoparticles on a screen-printed carbon electrode (Au/CuO/SPCE). The sensor's high sensitivity results from dual amplification strategies using AuNPs and CuO nanomaterials, each demonstrating catalytic activity towards glucose. This shows promising potential for saliva glucose monitoring. The AuNPs were synthesized using an Au precursor and orange peel extract (OPE), yielding stable colloidal AuNPs with a mean diameter of about 37 nm, thus eliminating the need for additional capping agents. Under optimal conditions, amperometric tests revealed that the sensor responded linearly to glucose concentrations ranging from 2 μM to 397 μM with a sensitivity of 236.70 μA mM^−1^ cm^−2^. Furthermore, the sensor demonstrated excellent reproducibility, stability and high selectivity for glucose in the presence of different biomolecules. We validated the sensor's efficacy by measuring glucose in human saliva, showing its potential for noninvasive glucose monitoring. This research advances the development of point-of-care devices, positioning the sensor as a promising tool for noninvasive glucose monitoring and improved diabetes management.

## Abstract 2

Gold nanoparticles (AuNPs) were synthesized using HAuCl_4_ and orange peel extract. AuNPs and CuO modified screen printed carbon electrode (Au/CuO/SPCE) converts glucose to gluconolactone. This sensor was applied for detection of glucose in saliva.

## Graphical abstract

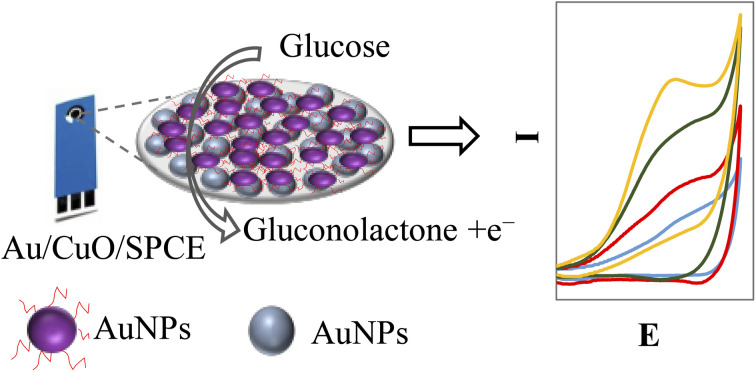

## 1 Introduction

Glucose is the primary energy source for cells and plays a vital role in brain and muscle function.^1,2^ Maintaining proper glucose levels is important for the functioning of the human body. However, abnormal levels of glucose can cause numerous life-threatening diseases, such as diabetes, hyperglycemia,^3^ and hypoglycaemia.^4^ These conditions can accelerate the development of various diseases, including but not limited to, type 2 diabetes, cardiovascular disease, stroke, kidney damage, nerve damage, and vision problems.^5,6^ According to WHO statistics, approximately 422 million adults worldwide suffer from diabetes.^7^ Regular monitoring of glucose is essential for early detection, tracking disease progression, and assessing treatment effectiveness. Moreover, monitoring glucose is crucial for analyzing aging behavior since diabetes mellitus in adults can lead to a decline in psychomotor speed. While both diabetic and nondiabetic individuals experience age-related cognitive declines, it remains unclear why diabetes impacts only certain cognitive skills.

Home-use glucometers have significantly improved the quality of life for patients with diabetes. However, most of these devices are enzymatic and require a finger prick to draw blood.^8,9^ Regular glucose monitoring is crucial for patients with diabetes mellitus to adjust insulin dosages for personalized medicine.^10^ Unfortunately, pricking the body multiple times a day can cause discomfort, leading to irregular blood sugar monitoring and reduced treatment effectiveness. Additionally, the use of needles incurs additional costs and poses risks of skin infections or other infectious diseases. While glucose monitoring using sweat and interstitial fluid is either noninvasive or minimally invasive,^11–13^ it can cause skin irritation and discomfort during the extraction of sweat or interstitial fluid. Consequently, noninvasive alternatives, such as saliva glucose monitoring, show great promise.

Three predominant methods, namely reducing, condensation, and enzymatic methods, are conventionally employed to measure blood glucose in laboratories. The reducing method involves the oxidation of glucose, leading to the reduction of a metal ion (for example, in Cu impedimetry) resulting in a color change of the analyte.^14^ In the condensation method, the aldehyde group in glucose undergoes a reaction with aromatic compounds, producing a colored product.^15^ However, due to the non-selectivity of the reducing method and the potential toxicity associated with the condensation method, these are not the preferred choices in clinical settings.^15^

There has been significant advancement in the detection of glucose using various enzymes, such as glucose oxidase (GOx),^16,17^ fluorescence immunoassay,^18^ and enzyme-linked immunosorbent assay (ELISA).^19^ However, nonenzymatic sensors are urgently needed to mitigate the problems associated with enzymatic sensors, such as high cost, instability, and susceptibility to environmental conditions.

Analytical techniques, exemplified by high-performance liquid chromatography (HPLC), are known to offer nonenzymatic glucose sensing.^20–23^ These methods enable the selective detection and quantification of an analyte based on its unique retention time and mass-to-charge ratio. However, they can be expensive, time-consuming, and require bulky equipment. Moreover, their operation often demands skilled personnel, particularly for complicated sample preparation, posing a significant challenge for routine on-site and real-time glucose monitoring.

Numerous nonenzymatic electrochemical glucose sensors have been reported, using metals,^24–26^ metal alloys,^27^ and metal oxides^28–30^ as they facilitate glucose oxidation without the use of enzymes. To enhance the sensing performance of metal or metal oxide-based glucose sensors, different composite materials have been introduced.^31^ However, these metal and metal oxide sensors are not suitable for saliva glucose monitoring (normal glucose range: 27.75 to 110 μM^32^) due to their inappropriate linear range. The performance of the metal and metal oxide sensors can be improved by combining them with different nanomaterials.^31,33–36^ However, carbon-based sensors show sensitivity towards common interferents present in biofluids, such as dopamine, uric acid, and ascorbic acid.^37,38^ Furthermore, the chemical synthesis process used for these sensors often involves the use of toxic, acidic, and strongly basic solvents or very high temperatures.^36^

Green analytical chemistry (GAC) aims to minimize the utilization of hazardous chemicals, employ energy-efficient equipment, and generate minimal waste, thereby promoting environmentally friendly practices.^39^ The different biochemicals present in the plant, flower or fruit extract work as reducing and capping agents, which offer stability and biocompatibility of synthesized gold nanoparticles. This necessitates further research on biomedical applications of AuNPs, including biosensing, drug delivery, imaging, and antibacterial applications.

On the other hand, copper oxide (CuO) is a very promising material for nonenzymatic glucose sensing. However, the linear range of such sensors does not suit saliva glucose monitoring.^28,40^ Gold nanoparticles (AuNPs), known for their biocompatibility, catalytic activity, and high stability, are extensively used in drug delivery, biomedical applications, and biosensing.^41,42^ Moreover, AuNPs themselves show an affinity towards glucose.^24,43^ A sensor using green synthesised AuNPs and graphene sheets has been reported for the detection of human immunodeficiency virus 1 (HIV-1) through DNA hybridization.^44^ The green synthesized AuNPs dispersed on graphene sheets that improved the attachment of DNA to the electrode and enhanced the sensitivity of the sensor.^44^ However, to the best of our knowledge, no research has been reported using green synthesized AuNPs for glucose sensing. Thus, developing a sensor that combines AuNPs with CuO could be beneficial for glucose sensing.

AuNPs can be synthesized through various methods, including colloidal synthesis, arrested precipitation, and ball milling.^45^ These processes often involve the use of toxic chemicals as reducing agents, capping agents, or solvents.^45^ Commonly used metal salt precursors include sodium borohydride,^46^ sodium citrate,^47^ and hydrazine sulphate.^48^ Given the inherent tendency of AuNPs to aggregate because of strong van der Waals forces, an additional capping agent is often necessary to stabilize their colloidal suspension.^49^ However, the use of chemicals, such as sodium borohydride, hydrazine sulfate, and other capping agents can increase fabrication costs, potentially exacerbate environmental pollution, and compromise the biocompatibility of AuNPs. This emphasizes the potential benefits of adopting green chemistry for AuNPs synthesis. Chloroauric (HAuCl_4_) salt is a commonly chosen precursor. With gold's high standard reduction potential (+0.994 V *vs.* SHE), a wide range of materials can effectively reduce HAuCl_4_ to Au(0).^50^ As a result, various non-toxic natural substances, including honey,^51^ starch,^52^ glucose,^52^ plant extracts,^53^ and orange peel extract (OPE),^54,55^ have been explored for this purpose. Given the ubiquity of oranges, we selected OPE as both a reducing and capping agent, aiming to repurpose waste effectively. While our investigation included the influence of parameters such as temperature, pH, and Au precursor concentration on the synthesis of AuNPs, it is noteworthy that some of these parameters were not exhaustively addressed in earlier studies.^51–55^

In this work, we developed a nonenzymatic glucose sensor using green-synthesized AuNPs and low-cost, wet-chemically synthesized CuO nanoparticles. Initially, we drop-coated CuO nanoparticles onto a screen-printed carbon electrode (SPCE). Then, we modified the CuO/SPCE with AuNPs. The Au/CuO/SPCE sensor exhibited a linear response range from 2 μM to 397 μM of glucose, with a sensitivity of 236.70 μA mM^−1^ cm^−2^, showing great promise for saliva glucose monitoring. We demonstrated the stability, reproducibility, selectivity and effectiveness of glucose detection in real saliva samples.

## 2 Experimental

### 2.1. Reagents

Chloroauric acid (HAuCl_4_), d-glucose, copper(ii) chloride, sodium hydroxide (NaOH), uric acid (UA), l-glutamic acid, ascorbic acid (AA), dopamine (DA), sodium phosphate dibasic (Na_2_HPO_4_), sodium phosphate monobasic (NaH_2_PO_4_), and Nafion™ 117 (approximately 5% in a mixture of lower aliphatic alcohols and water) were purchased from Sigma-Aldrich Canada. Potassium chloride (KCl), sodium chloride (NaCl), nitric acid (HNO_3_) and hydrochloric acid (HCl) were purchased from ACP Chemicals Canada. All these chemicals were of analytical grade and were used as received. A 0.1 M NaOH solution was used as the supporting electrolyte. Deionized (DI) water (with resistivity ≥ 18 MΩ cm) was used for preparing various aqueous solutions. A 100 mM glucose stock solution was prepared in DI water and stored in a refrigerator in a dark plastic bottle. Different concentrations of glucose were prepared by diluting the stock solution with 0.1 M NaOH. Suitable amounts of KCl, NaCl, Na_2_HPO_4_, and NaH_2_PO_4_ were dissolved in DI water to prepare 100 mM phosphate buffered saline (PBS) solutions of various pH, containing 2.7 mM KCl and 137 mM NaCl.

### 2.2. Apparatus

The PalmSens EmStat 3 Potentiostat was used for all electrochemical measurements. The SPCE, purchased from CH Instrument, comprised three printed electrodes: a 3 mm diameter carbon working electrode (area = 0.07 cm^2^), a carbon counter electrode, and an Ag/AgCl reference electrode. A 3D printed frame was used to secure the electrode, and a 70 μL drop of solution was placed onto the electrode for electrochemical measurement. Scanning electron microscopy (SEM) was performed using a JEOL 7100F microscope. UV-Vis spectroscopy was conducted using a Cary 5000 UV-Vis-NIR spectrophotometer. X-ray diffraction (XRD) analysis was carried out using the Bruker D8 DISCOVER instrument, utilizing Co Kα radiation (*λ* = 0.17902 nm) and scanning across the range of 2*θ* = 15°–105°, with a step size of 0.02°.

### 2.3. Green synthesis of AuNPs

The synthesis of AuNPs using OPE and HAuCl_4_ can be divided into two steps as follows.

#### Preparation of OPE

The orange was washed with DI water, and the peel was removed. We placed 40 gm of OPE and 100 mL of DI water into a round bottom (RB) flask equipped with a condenser. This mixture was heated to over 100 °C using an oil bath on a hotplate for an hour while stirring. After cooling to room temperature (RT, 20 °C ± 2 °C), the solution was filtered using Whatman grade 1 filter paper. The OPE was stored in a refrigerator at 4 °C to 8 °C for future use.

#### Synthesis of AuNPs

HAuCl_4_ was dissolved in DI water to a concentration range of 0.3–1 mM. 125 mL of the HAuCl_4_ solution was placed in an RB flask and heated it using an oil bath on a hotplate while stirring. When the solution reached the target temperature, such as 100 °C, we added 40 mL of OPE to the RB flask. The solution color quickly changed from light orange to dark purple, indicating the formation of AuNPs. We continued heating for an appropriate period, collecting samples at different times during the synthesis process to understand the reaction kinetics.

**Fig. 1:**
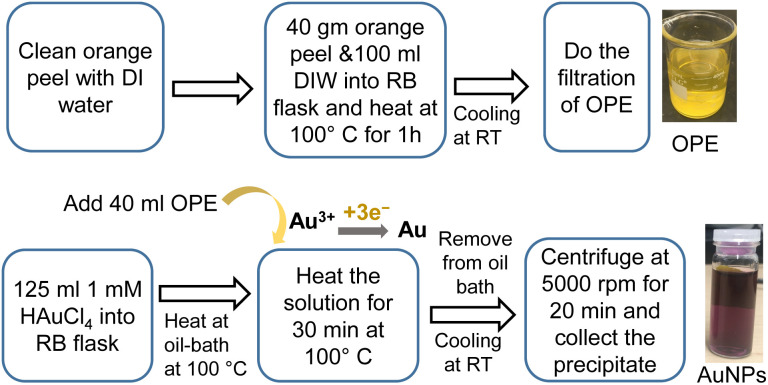
Green synthesis of AuNPs using HAuCl_4_ and OPE.

After the synthesis, the RB flask was removed from the oil bath and allowed to cool at RT. The solution was centrifuged at 5000 rpm for 20 min. The supernatant was discarded and an equivalent amount of DI water was added to the precipitate, followed by sonication for 2 min. After three washes, we stored the AuNPs dispersion in a refrigerator at 4 to 8 °C. Fig. 1 shows the schematic of the synthesis process of AuNPs using 1 mM HAuCl_4_ at 100 °C for 30 min.

**Fig. 2:**
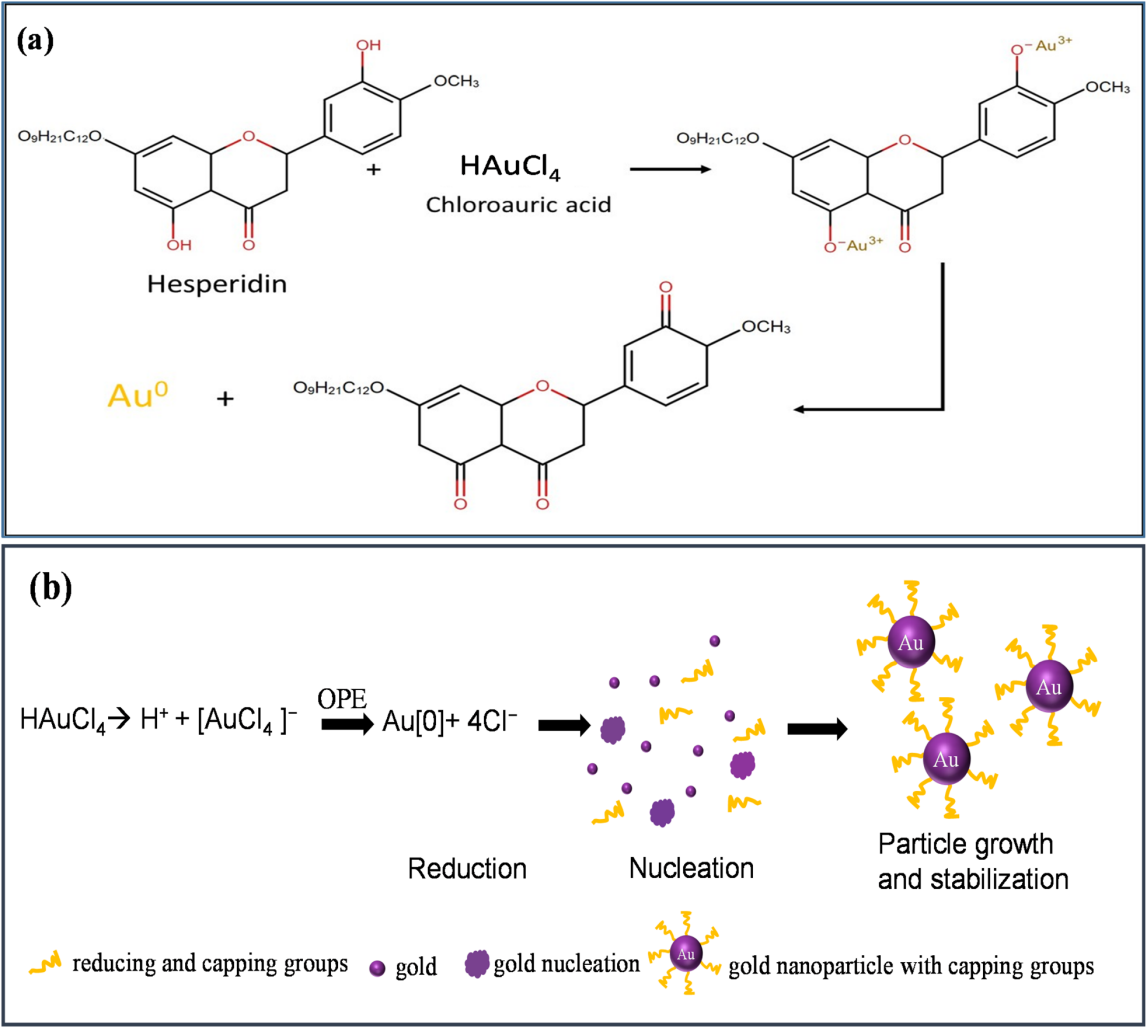
(a) Reduction of HAuCl_4_, (b) schematic of green synthesis mechanism of AuNPs using HAuCl_4_ and OPE.

OPE, rich in components like hesperidin, alcohol, phenolic compounds, amides, and esters, serves dually as reducing and capping agents.^56,57^Fig. 2(a) shows the reduction of HAuCl_4_ by hesperidin. The green synthesis mechanism for AuNPs involves several stages:

(i) reduction (ii) nucleation, and (iii) particle growth and stabilization, as depicted in Fig. 2(b).

When HAuCl_4_ is introduced into an aqueous medium, it dissociates into H^+^ and [AuCl_4_]^−^ ions. The subsequent introduction of OPE into this solution catalyzes the reduction of Au^3+^ to its elemental form. These elemental Au atoms, upon interaction, initiate nucleation. This nucleation phase leads to the evolution and growth of AuNPs over time. The process culminates with various capping agents interacting with and stabilizing the AuNPs.

### 2.4. Preparation of electrodes

We synthesized the CuO nanoparticles using a wet chemical process involving CuCl_2_ and NaOH, as detailed in ref. 58. To prepare a CuO dispersion of 5 mg mL^−1^, we dispersed 5 mg of CuO in 1 mL of DI water, followed by ultrasonication for 45 min. We then drop casted 8 μL of this CuO dispersion onto the working electrode of the SPCE and dried it at RT. To fabricate the Au/CuO/SPCE, we drop casted 4 μL of the green synthesized AuNPs dispersion onto the CuO/SPCE and dried at RT. The schematic diagram of the preparation of the Au/CuO/SPCE is shown in ESI (Fig. S1†). To enhance the adhesion of Au and CuO to the SPCE, we modified the Au/CuO/SPCE with 3 μL of 0.125% Nafion binder. We observed that a low concentration of Nafion does not affect the electrode's response.

## 3 Result and discussion

### 3.1. AuNPs characterization

#### Effect of HAuCl_4_ concentration and synthesis time

**Fig. 3:**
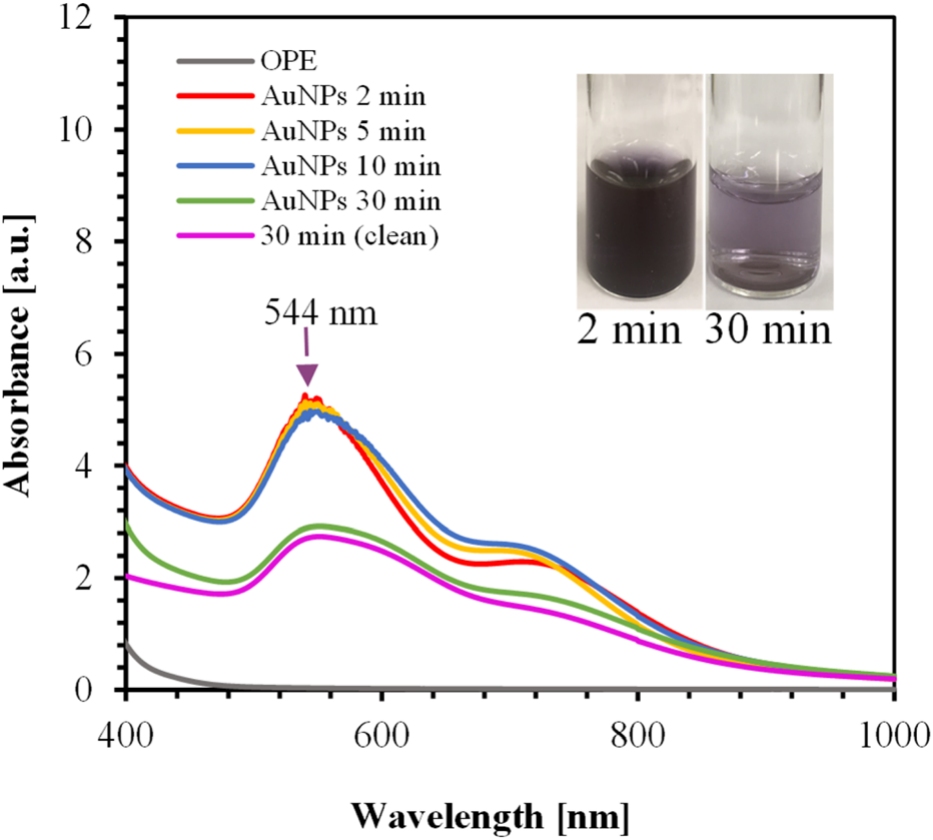
UV-Vis spectra of AuNPs synthesized over various times using 125 mL of 1 mM HAuCl_4_ and 40 mL of OPE. The inset shows the color of the AuNPs.

The Au precursor concentration as well as synthesis time have significant effect on size and throughput of AuNPs. We varied the HAuCl_4_ concentration as well as synthesis time, and collecting samples during synthesis to examine the reaction kinetics. Fig. 3 shows the UV-Vis spectra of AuNPs synthesized using 125 mL of 1 mM HAuCl_4_ and 40 mL of OPE for 30 min at 100 °C. The absorption peak at 544 nm indicates the formation of AuNPs. As seen in the figure, the reaction completed within 2 min as indicated by the red colored line. After 30 min of reaction time, a large number of AuNPs were deposited on the surface of the RB flask, resulting in a lower concentration of AuNPs in the solution and, correspondingly, a lower absorption peak.

OPE consists of different proteins, alcohols, phenolic compounds, carboxylic acids, esters, and ethers, as confirmed by FTIR analysis. These different functional groups in OPE act as capping agents.^54^ It is likely that heating at high temperatures for extended periods may alter the properties of these capping agents. The method of adding OPE to the HAuCl_4_ solution significantly impacts the stability of the AuNPs.

Adding OPE drop-wise to the HAuCl_4_ solution results in a second peak at 867 nm, as shown in ESI, Fig. S2.† In this process, orange peel serves as both a reducing agent and a capping agent. At high temperatures, it is likely that the nucleation and growth of AuNPs occur rapidly. Consequently, the drop-wise addition of OPE may not provide sufficient capping agents in the given time frame, leading to the aggregation of AuNPs. This aggregation causes the delocalization of conduction electrons, which are then shared among neighboring particles, resulting in a red shift at the surface plasmon resonance.^59^ For the drop-wise addition, the red shift relative to the first peak was approximately 317 nm.

Fig. S3(a) and (b) in ESI† shows the UV-Vis spectra of AuNPs synthesized using 0.5 mM and 0.3 mM HAuCl_4_, respectively. In all cases, the reaction completed within 2 min. The absorption spectra and the color changes of the AuNPs for different concentrations of HAuCl_4_ are shown in Fig. S3(c) and (d).† As shown in Fig. S3(c),† a small secondary peak appears at 718 nm for the 1 mM HAuCl_4_ solution, likely due to nanoparticle aggregation. Therefore, the optimum condition was identified as using 125 mL of 0.5 mM HAuCl_4_ with 40 mL OPE, where the color changes from pink to dark pink with increasing concentration of HAuCl_4_.

#### Effect of pH on AuNPs synthesis

We adjusted the pH of 125 mL DI water from pH 2.5 to 12 using HCl and NaOH, then added HAuCl_4_ to prepare a 0.5 mM HAuCl_4_ solution. The synthesis was conducted for 10 min at 100 °C. The impact of pH on AuNPs synthesis is shown in ESI, Fig. S4.† A strongly acidic medium is not favorable for stable AuNPs formation, while a strongly basic medium does not yield high throughput. AuNPs can be synthesized in a medium from weakly acidic to weakly basic (pH 5.5 to pH 9.1). We observed a dark purple color at pH 2.5 and pH 12 with a very dark purple hue between pH 5.5 and pH 9.1.

#### Effect of temperature on AuNPs synthesis

To examine the effect of temperature on AuNPs synthesis, we conducted experiments at various temperatures: RT (20 ± 2 °C), 60 °C, 75 °C, 90 °C, and 100 °C using 0.5 mM HAuCl_4_. We found that temperature had a noticeable impact on the synthesis of AuNPs. At RT, the synthesis was conducted for 18 hours with stirring. During this process, we collected samples of the AuNPs colloidal solution at different intervals, cleaned them, and analyzed them using UV-Vis spectroscopy to understand the reaction kinetics, as shown in ESI, Fig. S5(a).† After the addition of OPE to the HAuCl_4_ solution, a color change to dark pink was observed within 15 min, becoming more pronounced after 2 hours, as shown in Fig. S5(b).† However, we observed agglomeration of AuNPs at the bottom of the vial after 2 days for the 2 hours synthesis, and after 5 days for the 18 hours synthesis. The nucleation and growth using OPE is a rapid process even at RT due to the high reduction potential of AuCl_4_^−^/Au(0). Nonetheless, the interaction between Au(0) and the capping agents in the OPE appears to be slower and non-spontaneous process at RT, leading to the aggregation of AuNPs.

**Fig. 4:**
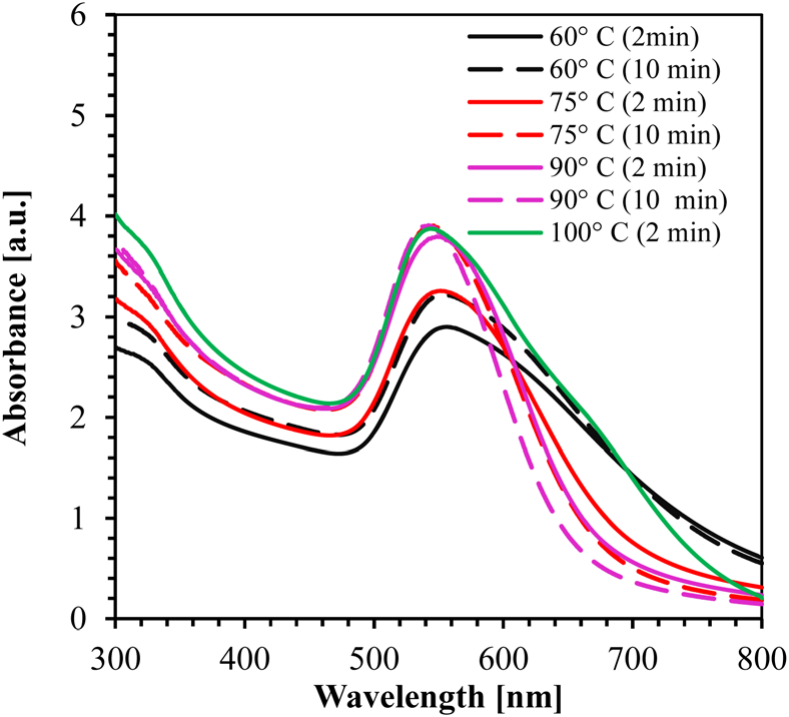
UV-Vis absorption spectrum of AuNPs synthesized at different temperatures using 0.5 mM HAuCl_4_.

The UV-Vis absorption spectrum for 60°, 75°, 90°, and 100 °C is shown in Fig. 4. From the figure, it is clearly observed that the synthesis at temperature ≥ 90 °C for 10 min yielded the smallest NPs (peak at 542 nm wavelength) with high absorption peaks.

#### FTIR spectroscopy

**Fig. 5:**
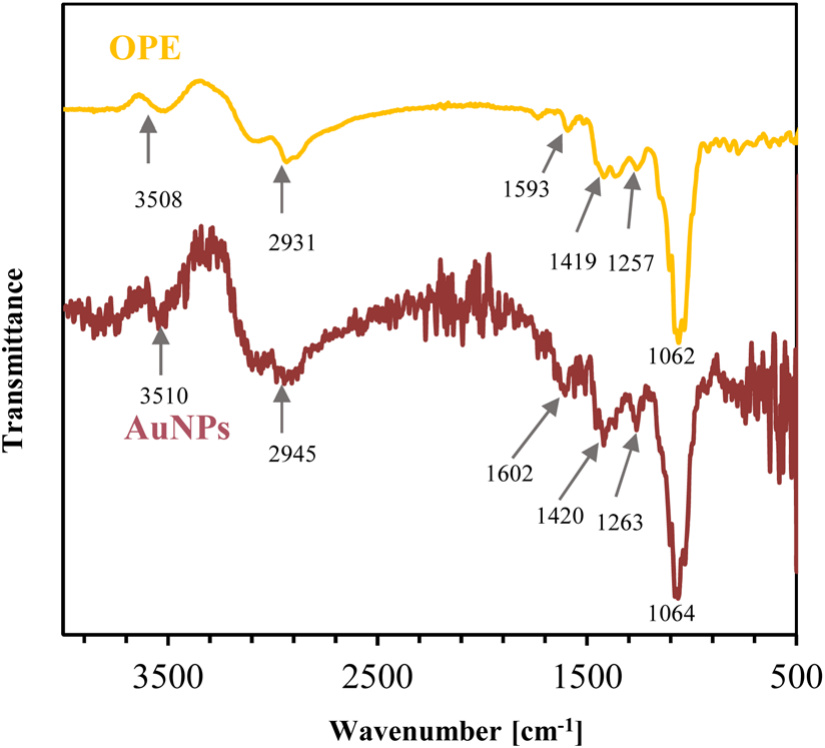
FTIR spectra of OPE in liquid form and clean AuNPs in colloidal form, synthesized using 0.5 mM HAuCl_4_ at 90 °C for 10 min.

Stable nanoparticles provide high effective surface area which can increase the sensitivity of the sensor. FTIR spectroscopy was used to identify the functional groups and biochemical compounds present in the OPE and green synthesized AuNPs, which are instrumental in stabilizing the AuNPs. Fig. 5 shows the FTIR spectra of both the OPE and the cleaned AuNPs.

OPE shows intense absorption bands at 3508, 2931, 1593, 1419, 1257, and 1062 cm^−1^. In the FTIR spectra, AuNPs display noticeable bands at 3510, 2945, 1602, 1420, 1263, and 1064 cm^−1^. The bands around 3508 and 3510 cm^−1^ are O–H stretching vibrations in alcohol and phenolic compounds,^60^ and bands around 2931 and 2945 cm^−1^ are C–H stretching in the methyl group.^61^ Bands at 1593 and 1602 cm^−1^ are due to C=O stretching (the amide I band), which is greatly influenced by secondary structure of the protein.^62^ Peaks at 1419 and 1420 cm^−1^ are associated with C–C stretching in alcohol, carboxylic acid, ester and ether.^63^ The bands at 1275 and 1263 cm^−1^ result from N–H bending and C–H stretching (amide III band), correlated to the protein's secondary structure. The peaks around 1062, 1064 cm^−1^ are associated with C–O stretching in alcohols and phenols.^64^

#### X-ray diffraction (XRD) and SEM analysis

**Fig. 6:**
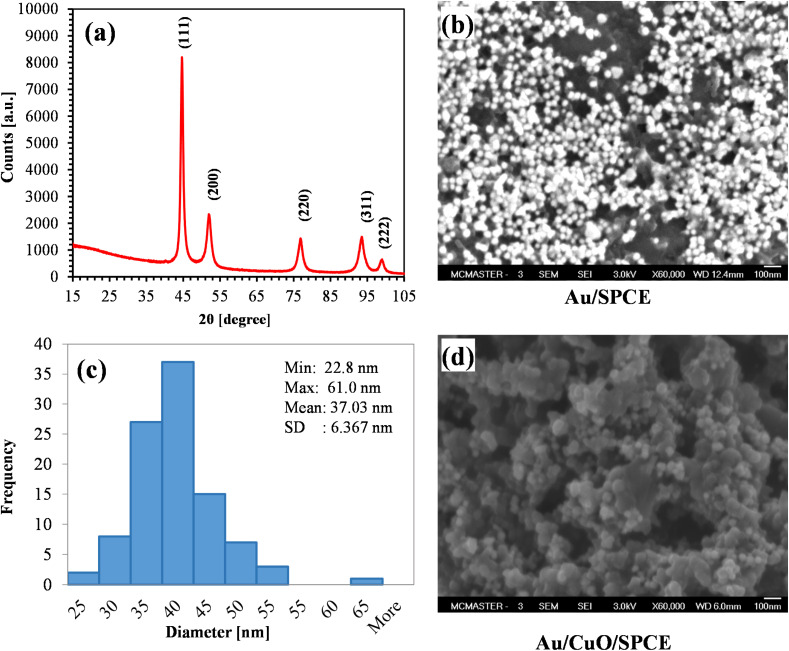
(a) XRD spectra of AuNPs (pattern: PDF 03-065-8601); (b) SEM image showing AuNPs on SPCE; (c) size distribution of green synthesized AuNPs (for 100 NPs); (d) surface morphology of Au/CuO/SPCE.

We examined the crystalline nature of the synthesized AuNPs using 0.5 mM HAuCl_4_ through XRD, as presented in Fig. 6(a). The peaks at 2*θ* = 44.72, 52.12, 76.82, 93.53 and 99.09 correspond to the Bragg reflections (111), (200), (220) (311), and (222), respectively, according to the ICDD database PDF # 03-065-8601. The most intense peak, occurring at 44.72 is attributed to the (111) facets of face-centered cubic metal Au structures, indicating that the AuNPs synthesized from 0.5 mM HAuCl_4_ and orange peel at 90 °C are crystalline Au.

The SEM image of AuNPs on the SPCE electrode is shown in Fig. 6(b). The figure clearly illustrates that the synthesized AuNPs are spherical in shape. Most of AuNPs are separate, indicating that the synthesis process yielded very stable AuNPs. We calculated the size of 100 AuNPs using ImageJ software. The size distribution of AuNPs is shown in Fig. 6(c). Most of the particle size belong the range of 30 to 40 nm, with a mean diameter of approximately 37 nm. Fig. 6(d) shows the surface morphology of the Au/CuO/SPCE, revealing a very rough surface with the AuNPs nicely covering CuO surface.

### 3.2. Role of materials for glucose sensing

To examine the electrochemical performance of AuNPs synthesized using 0.5 mM HAuCl_4_ at different temperatures, we prepared different electrodes by modifying SPCE with 8 μL of AuNPs using drop casting. We used 5 mM K_4_[Fe(CN)_6_] with 100 mM KCl as a redox probe. The cyclic voltammetry (CV) responses of those electrodes are shown in ESI, Fig. S6.† The current density (*J*) is calculated by dividing the current (*I*) by the electrode area (*A* = 0.07 cm^2^). All AuNPs electrodes exhibited a higher current than the bare SPCE which is attributed to the high conductivity and effective surface area of the AuNPs/SPCE. AuNPs act as a catalyst for the oxidation of K_4_[Fe(CN)_6_], providing higher currents and reducing the oxidation voltage by 40 mV compared to bare SPCE (*E*_pa_ = 280 mV). It is clearly observed from the results that AuNPs synthesized at higher temperatures perform better than those synthesized at lower temperatures. Thus, for glucose sensing, we used AuNPs synthesized at 100 °C for 10 min.

**Fig. 7:**
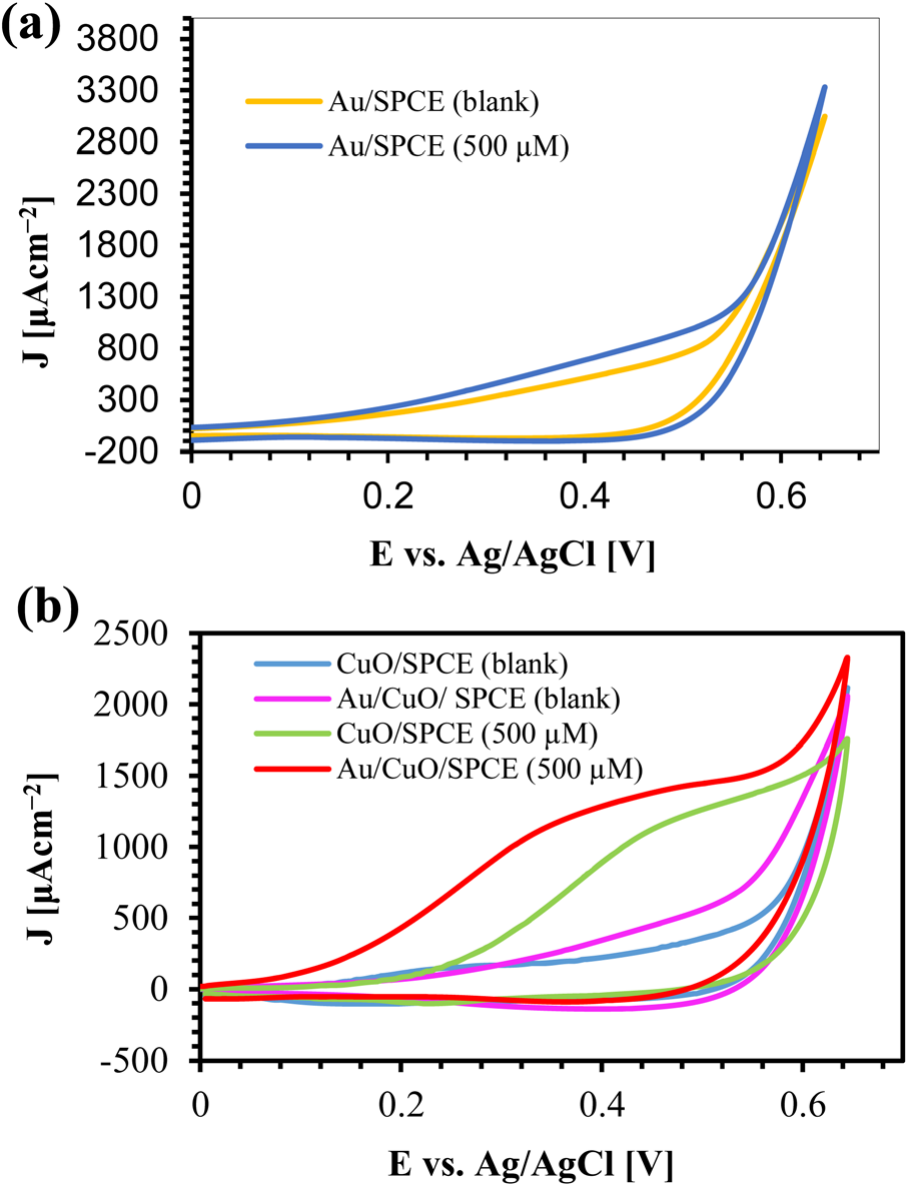
CV responses of (a) Au/SPCE and (b) CuO/SPCE and Au/CuO/SPCE with glucose and 0.1 M NaOH at a scan rate 60 mV s^−1^. “Blank” refers to 0.1 M NaOH.

The CV responses of different electrodes in the presence of 500 μM glucose with 0.1 M NaOH are shown in Fig. 7, where *J* represents the current density. Au/SPCE offers a higher current than the other electrodes in the blank (0.1 M NaOH), indicating the good conductivity of AuNPs. The addition of 500 μM glucose results in a slight increase in electroactive area (Fig. 7(a)), which is attributed to the catalytic activity of Au towards glucose.^65,66^ From the XRD, we observed the most extensive peak from Au (111). The adsorption of OH^−^ from the electrolyte and H_2_O converts Au (111) to AuOH, which oxidizes glucose to produce gluconolactone. This gluconolactone is then rapidly hydrolized to gluconic acid.^67^

**Fig. 8:**
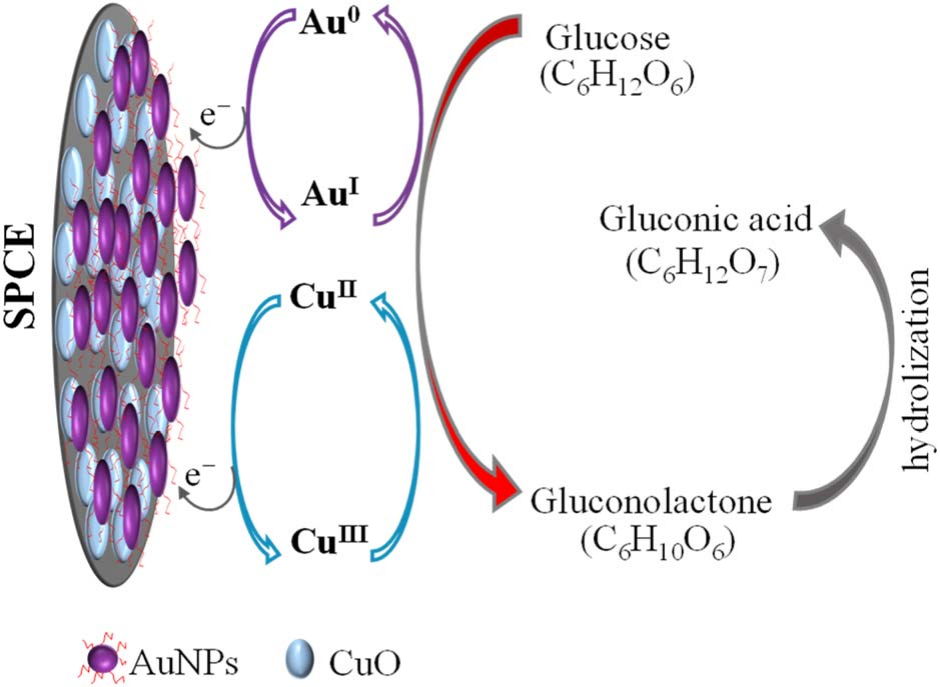
Electro-oxidation process of glucose on the AuNPs/CuO/SPCE surface in a NaOH supporting electrolyte. The applied potential induces the conversion of Au(0) to Au(i) and Cu(ii) to Cu(iii), facilitating the oxidation of glucose to gluconolctone, which subsequently converts to gluconic acid.

CuO/SPCE exhibits good catalytic activity towards glucose, where the oxidation voltage (*E*_pa_) is about 0.5 V. The sensing mechanism of glucose using CuO in an alkaline medium is well established.^28,68^ In alkaline conditions, CuO is electrochemically oxidized and converted to a Cu(iii) compound, such as, CuOOH and Cu(OH)_4_^−^. In the presence of Cu(iii), glucose is oxidized and converted into gluconolactone while Cu(iii) is simultaneoulsy reduced to CuO/Cu(OH)_2_.^28,68^ Ultimately, the gluconolactone is oxidized to form the stable product, gluconic acid through hydrolyzation process. When CuO used as an electrode, it facilitates the conversion from Cu(ii) to Cu(iii) at high potentials (above 0.7 V).^28^ However, the use of CuO nanoparticles enables the oxidation of glucose at a reduced potential, approximately 0.5 V, as shown in Fig. 7(b). Modifying CuO/SPCE with AuNPs further increases the oxidation current and reduces the *E*_pa_ to 0.4 V. This enhancement can be attributed to the synergistic electrochemical catalytic activities of AuNPs and CuO. The sensing mechanism of glucose at the Au/CuO/SPCE electrode is shown schematically in Fig. 8.

### 3.3. Performance optimization of Au/CuO/SPCE

The current response to glucose influenced by several factors, including the amounts of CuO and AuNPs drop-coated on the electrode, the scan rate, NaOH concentration, and the range of sweeping potentials. Optimum results are obtained by drop-casting 8 μL of 5 mg per mL CuO, 4 μL of AuNPs, and 3 μL of 0.125% Nafion, using 0.1 M NaOH as the supporting electrolyte and a scan rate of 60 mV s^−1^. For each electrochemical measurement, we consistently use 70 μL of the solution to cover the electrode surface and allow 3 min for the interaction between the analytes and the electrode surface.

### 3.4. Effect of NaOH concentration and scan rate

We used PBS with varying concentrations of NaOH to examine the impact of NaOH concentration on CuO oxidation. In glucose sensing using a CuO-based electrode, the sensing activity depends on the formation of a Cu(ii) to Cu(iii) compound.^28,68^ As shown in Fig. S7(a),† the NaOH electrolyte plays a significant role in the oxidation of CuO. However, PBS alone, even with pH 10 cannot oxidize CuO. The oxidation of CuO requires NaOH, and the extent of CuO oxidation increases with the concentration of NaOH. High concentrations of NaOH are favourable for glucose oxidation, as shown in Fig. S7(b).†

The effect of scan rate on glucose sensing was examined using Au/CuO/SPCE by varying the scan rate from 20 to 120 mV s^−1^ in the presence of 1 mM glucose with 0.1 M NaOH. The CV responses at different scan rates are shown in ESI, Fig. S8(a).† The oxidation current of glucose increases with the scan rate. From Fig. S8(b),† the linear relationship between *I*_pa_ and *ν*^1/2^ can be expressed as:

1 *I*_pa_ (μA) = 16.073*ν*^1/2^ − 35.956 (*ν*: mV s^−1^, *R*^2^ = 0.9933)

This linear relationship between the current and the square root of the scan rate indicates that the mass transport at the electrode is a diffusion-controlled process.

### 3.5. Detection of glucose

**Fig. 9:**
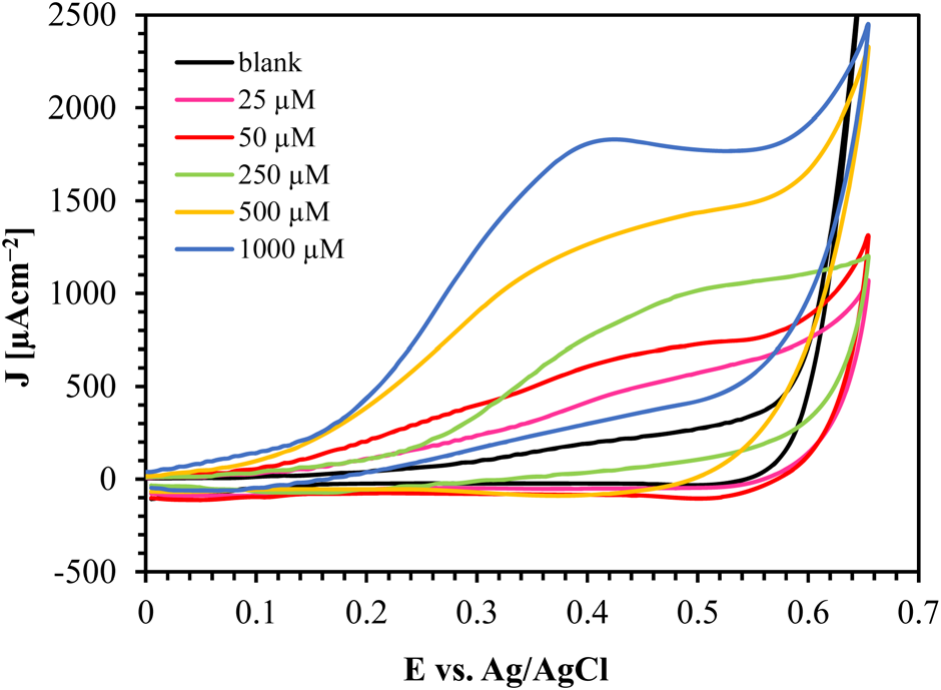
CV responses of Au/CuO/SPCE at varying glucose concentration in 0.1 M NaOH at a scan rate of 60 mV s^−1^.

The CV results of Au/CuO/SPCE with different glucose concentrations are shown in Fig. 9. This sensor oxidizes glucose at a relatively low potential and offers a high oxidation current, providing a substantial oxidation current even at low glucose concentrations (25 μM).

**Fig. 10:**
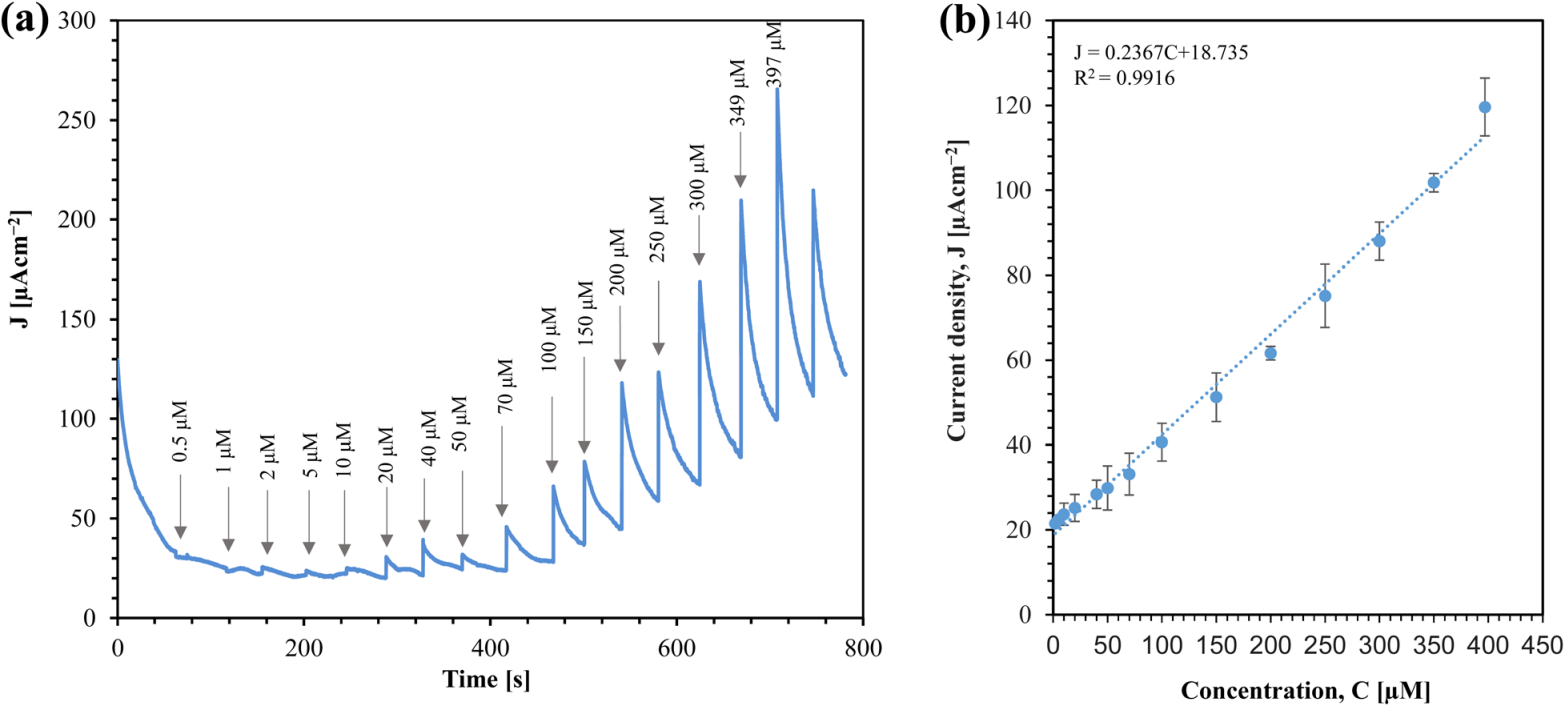
(a) Amperometric response of Au/CuO/SPCE to different glucose concentrations at 0.4 V. The arrow indicates the total glucose concentration after each addition. (b) Calibration plot with standard deviation for triplet measurements.

From a practical standpoint, the implemention of amperometric sensor is straightforward, as it operates with a constant voltage. We investigated the performance of the sensor for glucose sensing using amperometry, as shown in Fig. 10(a).

From Fig. 10(a), it is clear that the sensor's response increases with the concentration of glucose. The corresponding calibration plot for the sensor is shown in Fig. 10(b). The sensor offers a linear range from 2 μM to 397 μM. The sensitivity of the sensor is determined by the slope of the current density (*J*) *vs.* concentration (*C*) graph. Thus, the sensitivity of the sensor is 0.23670 μA cm^−2^ μM^−1^, which is equivalent to 236.70 μA cm^−2^ mM^−1^. This linear range makes the sensor promising for real-time monitoring of saliva glucose.^32^

The calibration equation can be expressed as:

2 *J* (μA cm^−2^) = 0.2367*C* (μM) + 18.735 [*C* = 2 to 397 μM]

We calculated the limit of detection (LOD) (7.24 μM) of the sensor using the formula LOD = 3*σ*/*m*, where *σ* is the standard deviation of the blank response (0.5714 μA cm^−2^) and *m* is the slope of the calibration curve. Table 1 compares this work with conventional chemically synthesized and green synthesized sensors. While the sensitivity of the sensor in this work is comparable with other sensors, it outperforms others in terms of the linear range, especially for saliva glucose monitoring.

**Table 1:** Comparison of green synthesized AuNPs based sensor in this work with other conventional chemically synthesized sensors^a^

Electrode	LOD (μM)	Linear range	Sensitivity (μA cm^−2^ mM^−1^)	Real sample	Ref.
**Conventional chemically synthesized sensor**					
AuNW/glass	50	1–10 mM	309.00	—	24
PtAu–MnO_2_/GP	20	100 μM–30 mM	58.54	Urine, serum	69
CuNO_*x*_	94.2	50 μM–7 mM	603.42	—	70
Ni@CN/3D-KSCs	7.85	24 μM–1.2 mM	—	Blood	31
NF/CuO NF/GCE	0.2	100 μM–10.8 mM	483.10	—	30
Ni–CuO/Cu/Cu_2_O	0.7	10 μM–8 mM	208.50	Serum	71

**Green synthesized sensor**					
npGNPs/C	0.1	1–50 μM	6670	—	72
rGO–AuNPs/GCE	10	18 mM	—	Serum	73
CuO/rGO-PGE	0.09	0.1–150 μM	4760	Serum	74
NiONPs/GCE	3.2	10–200 μM	—	—	75
Pd–CSP/C	237	1–8 mM	17.7	Serum	76
ZnO@NDCS/GCE	6.3	200 μM–12 mM	231.7	Serum	77
**g-Au/CuO/SPCE**	**7.24**	**2–397 μM**	**236.70**	**Saliva**	**This work**

a g-Au: green synthesized gold nanoparticles, AuNW: gold nanowires, GCE: glassy carbon electrode, CuO NF: copper oxide nanofiber, CuNO_*x*_: native copper oxide, Ni@CN/3D-KSCs: Ni@carbon nanocomposites/three-dimensional kenaf stem, PtAu–MnO_2_/GP: platinum gold bimetal-manganese-oxide/graphene paper, Ni–CuO: Ni doped CuO, npGNPs/C: nanoporous gold nanoparticles/carbon, rGO-AuNPs: green synthesized reduced graphene oxide decorated with gold NPs, CuO/rGO–PGE: green CuO on rGO pencil graphite electrode, NiONP: nickel oxide nanoparticle, Pd–CSP/C: Pd NPs using *Cynomorium songaricum* polysaccharide, ZnO@NDCS: zinc oxide (ZnO) nanoparticles embedded nitrogen doped carbon sheets.

### 3.6. Stability, reproducibility and interference studies

We tested the reproducibility of the sensor by fabricating a batch of 10 samples of Au/CuO/SPCE and measuring the CV response in the presence of 1 mM glucose, as shown in ESI, Fig. S9(a).† The sensor showed good reproducibility with a low relative standard deviation (RSD, 4.86%). To examine the stability of the sensor, we prepared Au/CuO/SPCE and exposed it to open air at RT. On different days, we measured the CV response using a new electrode each time, in the presence of 1 mM glucose in 0.1 M NaOH at a scan rate of 60 mV s^−1^, as shown in ESI, Fig. S9(b).† While the oxidation potential drifted towards a lower value over time, the oxidation current at 0.4 V remained nearly the same. Given that CuO nanomaterials are sensitive to humidity and temperature, this may explain the shift in oxidation potential towards a lower value. Remarkably, the sensor maintained a reasonable response even after 3 weeks from synthesis.

**Fig. 11:**
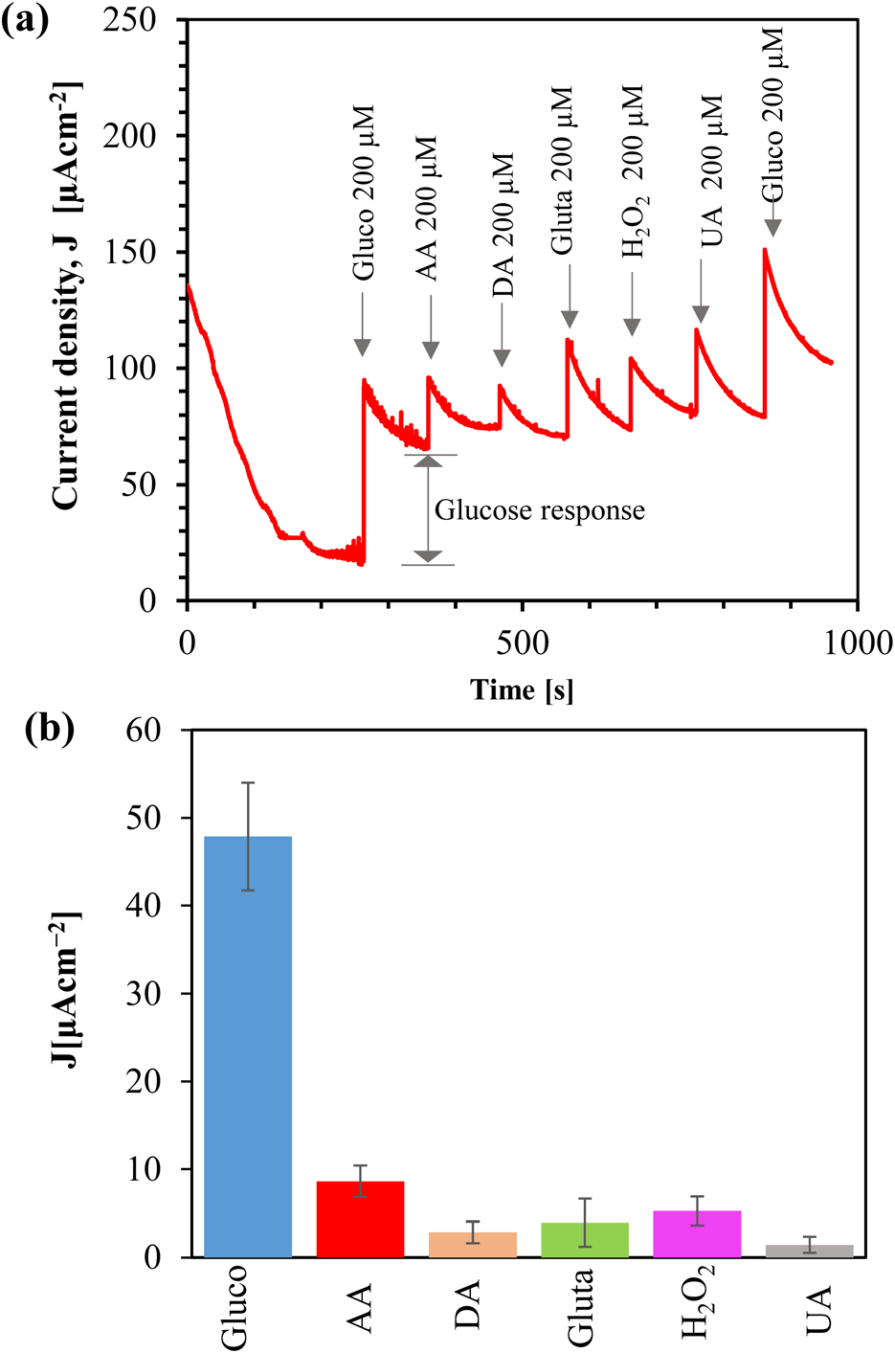
(a) Amperometric response of Au/CuO/SPCEs to various analytes in 0.1 M NaOH at 0.4 V; (b) bar plot showing current density responses different analytes (Gluco: glucose, AA: ascorbic acid, DA: dopamine, Gluta: glutamate, H_2_O_2_: hydrogen peroxide, and UA: uric acid).

We examined the selectivity of the Au/CuO/SPCE by adding different interferent species, such as ascorbic acid (AA), dopamine (DA), glutamate (gluta), and uric acid (UA), which are commonly present in biofluids. Amperometry was conducted at 0.4 V using a supporting electrolyte of 0.1 M NaOH. After the current stabilized, we introduced 200 μM of glucose. Subsequently, various interfering species were addedd, and followed by another addition of glucose, as shown in Fig. 11(a). Before introducing each analyte, we temporarily paused the amperometry and immediately added the analyte and resumed the measurement. After adding the analyte, we stirred the sample magnetically for 15 seconds to mix it, waited for another 15 seconds without stirring, and then continued the amperometry for 100 seconds. The final current measured at the end of these 100 s of amperometry is considered the response due to the addition of the analyte, as shown in Fig. 11(a).

The oxidized product of glucose tends to foul the electrode, which hinders the diffusion of glucose toward the electrode, and thus, over time, the current decreases. Stirring serves two functions: (i) it mixes the sample with the analyte, and (ii) it helps remove the oxidized product from the electrode surface. Although the initial current spiked after adding the interferents due to stirring, the actual response of these substances was low, as shown in the bar plot (Fig. 11(b)). We added 200 μM of each interfering analyte, however, the concentration of these analytes in the saliva is typically very low.^78–81^ Therefore, the sensor demonstrates high selectivity toward glucose.

### 3.7. Real sample analysis

**Fig. 12:**
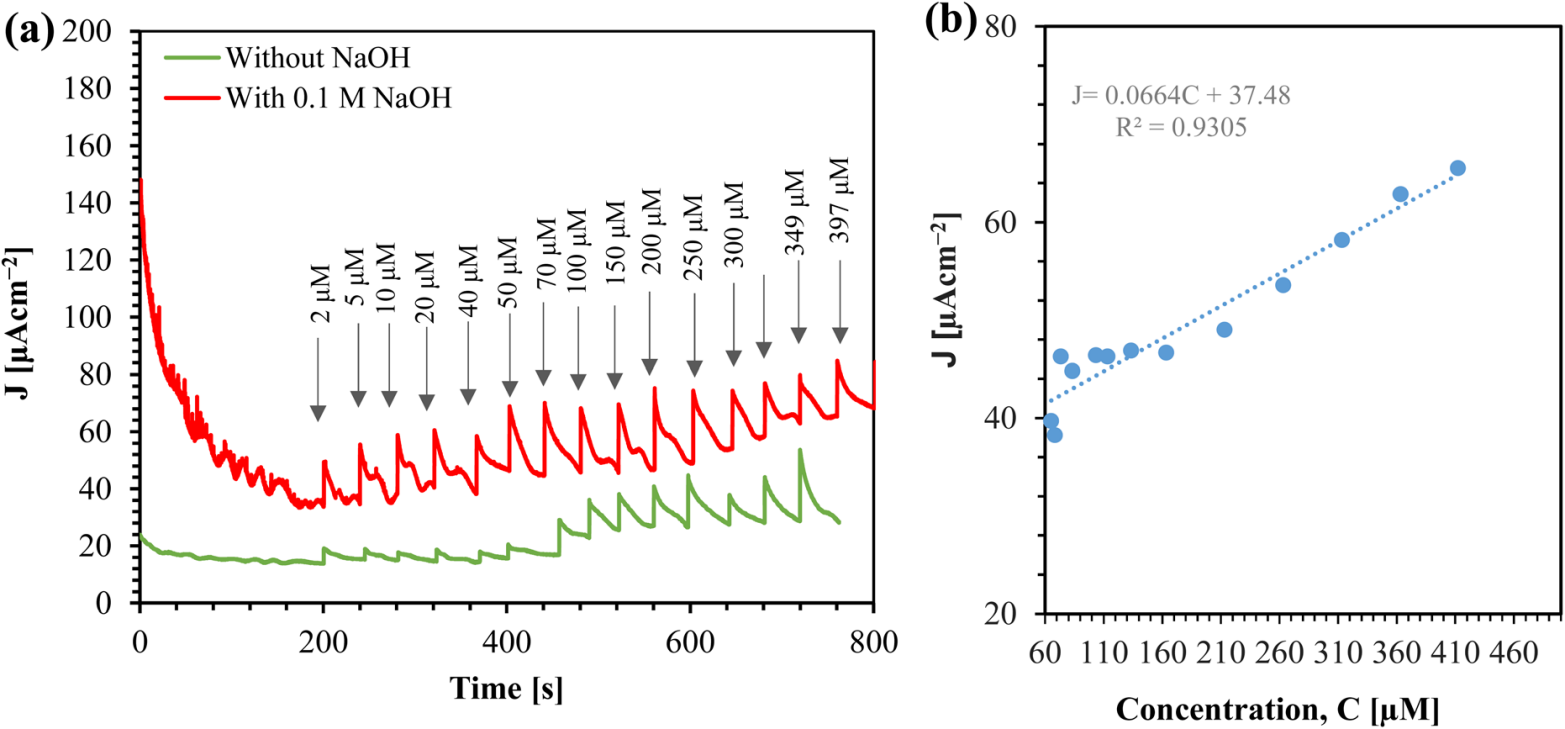
(a) Amperometric response of Au/CuO/SPCE in human saliva spiked with glucose at 0.4 V (arrow indicates the total glucose concentration added). (b) Calibration curve for glucose detection in saliva using Au/CuO/SPCE in 0.1 M NaOH.

We tested the efficacy of the sensor by detecting glucose in human saliva. The saliva was collected, stored at 4 °C, and analyzed the glucose within 4 hours of sample collection. Amperometry was performed on the saliva both with and without the addition of supporting electrolyte, and in both cases, the saliva was spiked with glucose, as shown in Fig. 12(a). An alkaline medium aids in the oxidation of CuO, which facilitates the conversion of glucose to gluconolactone.^28^ Therefore, in the absence of NaOH, the sensitivity in saliva is not optimal. However, adding NaOH to the saliva significantly increases the current.

Before the saliva was spiked with glucose, the current density (*J*) measured 33.842 μA cm^−2^. Using this value, we calculated the glucose level in the saliva to be 63.19 μM, based on calibration eqn (2). Fig. 12(b) presents the calibration curve for glucose detection in saliva. To obtain the calibration equation, we addedd the detected glucose level (63.19 μM) to the spiked glucose level. The sensitivity of the sensor for glucose sensing in real saliva samples was determined to be 66.48 μA mM^−1^ cm^−2^.

## 4 Conclusions and perspectives

In this work, we developed an innovative, facile, and highly sensitive non-enzymatic glucose sensor using green-synthesized gold nanoparticles (AuNPs) and wet chemically synthesized copper oxide (CuO) nanoparticles. The distinctive feature of this sensor is its ability to non-invasively monitor glucose levels in saliva. The AuNPs are synthesized using orange peel extracts, ensuring uniformity in size and increased stability. SEM images indicate that the AuNPs have a mean diameter of 37 nm, consistent with findings from other scientific techniques. Additionally, XRD studies reveal crystalline Au structures.

The sensor's high sensitivity can be attributed to the synergistic electrochemical activities of the AuNPs and CuO. This device can detect glucose concentrations ranging from 2 μM to 397 μM with a sensitivity of 236.70 μA mM^−1^ cm^−2^. Furthermore, it demonstrates excellent reproducibility (RSD = 4.86%), maintains stability for up to 3 weeks, and retains selectivity even the presence of other interfering species in human biofluids. The performance of this novel sensor can be fine-tuned through various factors, such as temperature, HAuCl_4_ concentration, pH, synthesis time, and post-synthesis cleaning, which substantially influence the stability, throughput, and electrochemical properties of the AuNPs. The green synthesis process, which is carried out at ambient temperature, enhances the sensor's eco-friendliness and cost-effectiveness. The broader implications of this work are significant, resonating with several Sustainable Development Goals.^82,83^ Utilizing green analytical chemistry and sustainable materials not only promotes research in scientific fields but also elevates the quality of education and fosters technological growth and innovation.

## Author contributions

M. Y. A.: conceptualization, data curation, formal analysis, investigation, validation, writing—original draft, review and editing. H. B. A.: conceptualization and formal analysis. W. T. T.: data curation and validation. M. M. R. H.: conceptualization, review and editing, funding acquisition and supervision. All authors have read and agreed to the published version of the manuscript.

## Conflicts of interest

There are no conflicts to declare.

## Supplementary Material

RA-014-D3RA05644A-s001
