# Supplementary material for: Green synthesized gold nanoparticles and CuO-based nonenzymatic sensor for saliva glucose monitoring
Source: RSC Adv. 2024 Jan 2;14(1):577–88. doi: 10.1039/d3ra05644a (PMC10758929; doi:10.1039/d3ra05644a)
Supplement: RA-014-D3RA05644A-s001 [file RA-014-D3RA05644A-s001.pdf]

Electronic Supplementary Material (ESI) for RSC Advances.
This journal is © The Royal Society of Chemistry 2023

Green synthesized gold nanoparticles and CuO-based nonenzymatic electrochemical sensor for saliva glucose monitoring

Md Younus Ali[a], Heman B. Abdulrahman[a], Wei-Ting Ting[a], Matiar M. R. Howlader[a]*

Department of Electrical and Computer Engineering, McMaster University

1280 Main Street West, Hamilton, ON, L8S 4K1, Canada

*Correspondence: howladm@mcmaster.ca

Supplementary Information

Green synthesis process for gold nanoparticle (AuNPs)

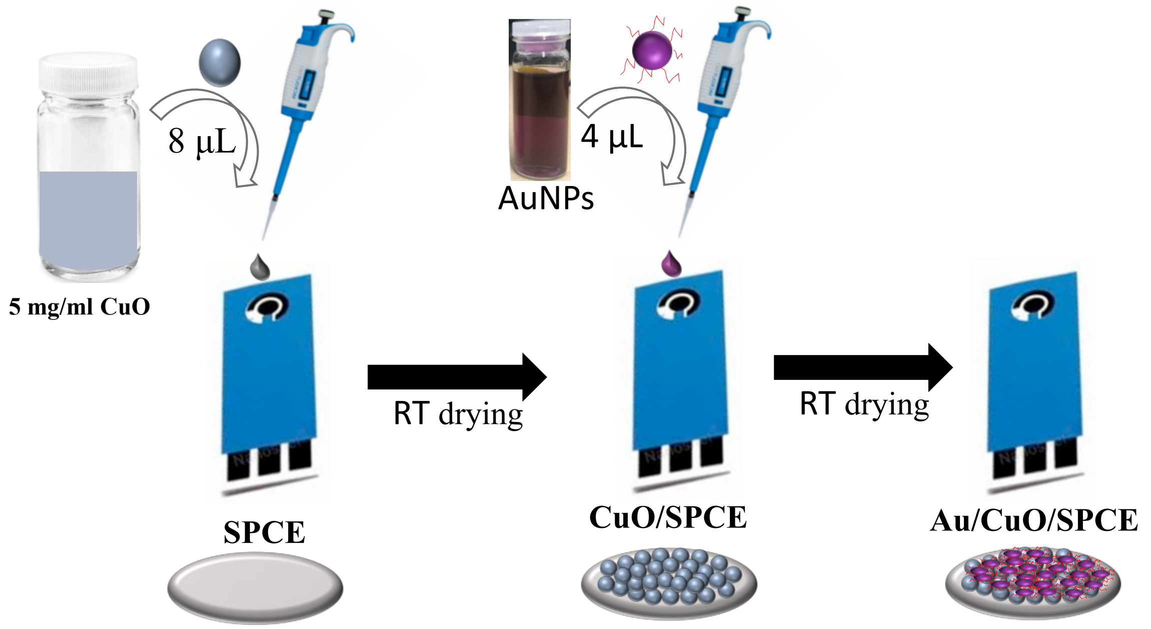

Fig.S1 . Schematic of preparation of Au/CuO/SPCE

AuNPs synthesis

Effect of precursor concentration and synthesis time

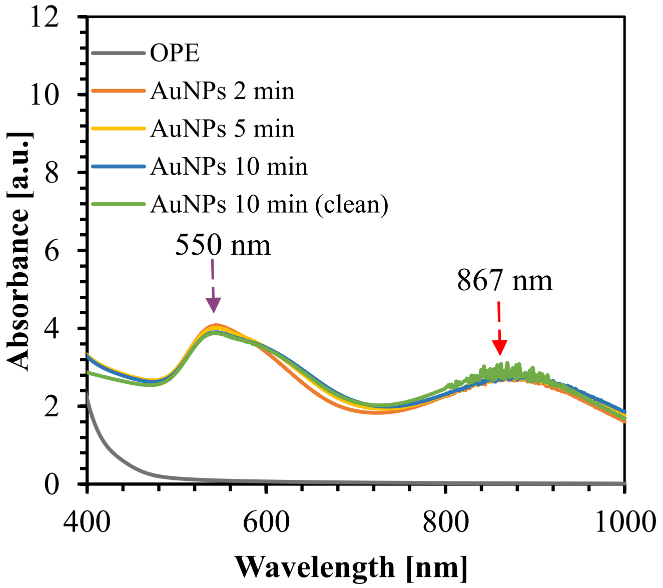

Fig. S2. UV-Vis absorption spectra of AuNPs using 125 ml of 1 mM $HAuCl_4$ where 40 ml of orange peel extract (OPE) were added drop-wise.

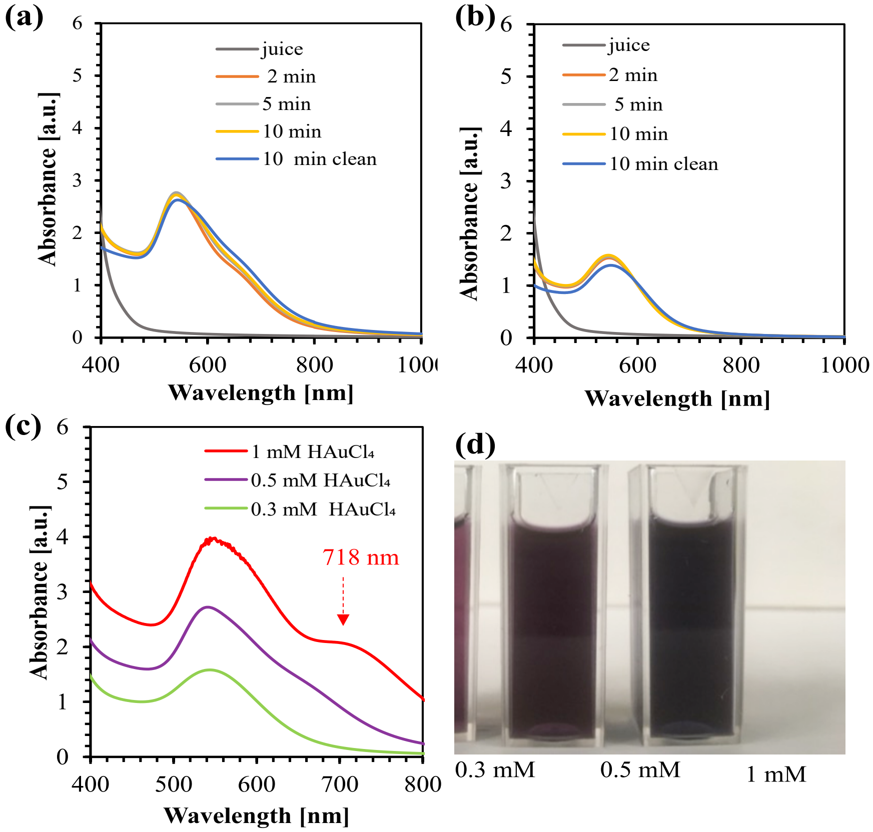

Fig. S3. UV-Vis absorption spectrum of AuNPs synthesis from (a) 0.5 M $HAuCl_4$, (b) 0.3 M $HAuCl_4$ at 100° C, (c) adsorption spectra of AuNPs for different concentration of $HAuCl_4$ for two minutes synthesis, (d) color after 10 minutes synthesis (from pink to dark pink).

Effect of pH on AuNPs synthesis

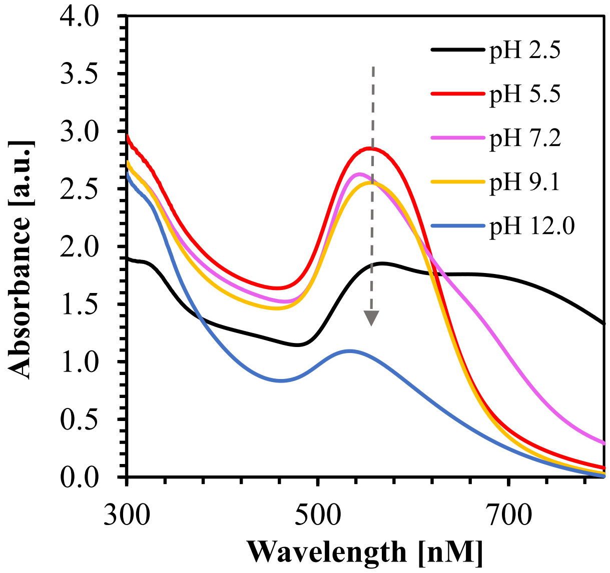

Fig.S4. UV-Vis absorption spectrum of AuNPs synthesis using 125 ml 0.5 mM $HAuCl_4$ and 40 ml OPE at 100° C for 10 min with different pH.

Effect of temperature on AuNPs synthesis

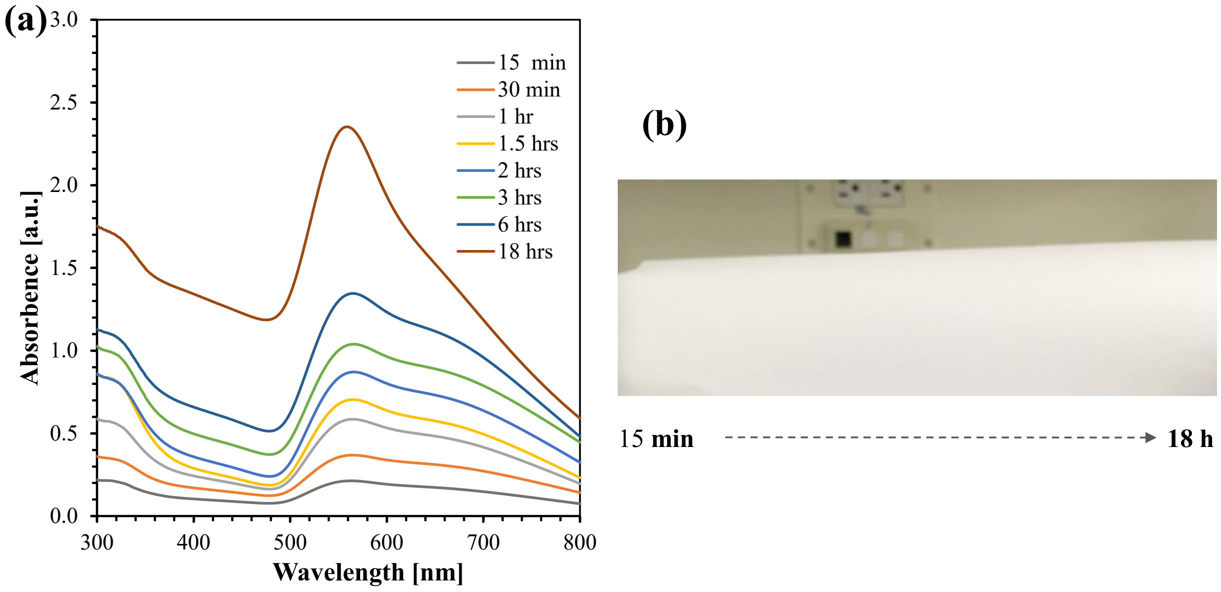

Fig. S5. Room temperature synthesis of AuNPs using 0.5 mM $HAuCl_4$, (a) UV-Vis absorption spectrum and (b) AuNPs' colloidal color at different synthesis time.

Electrochemical performance of Au/SPCE

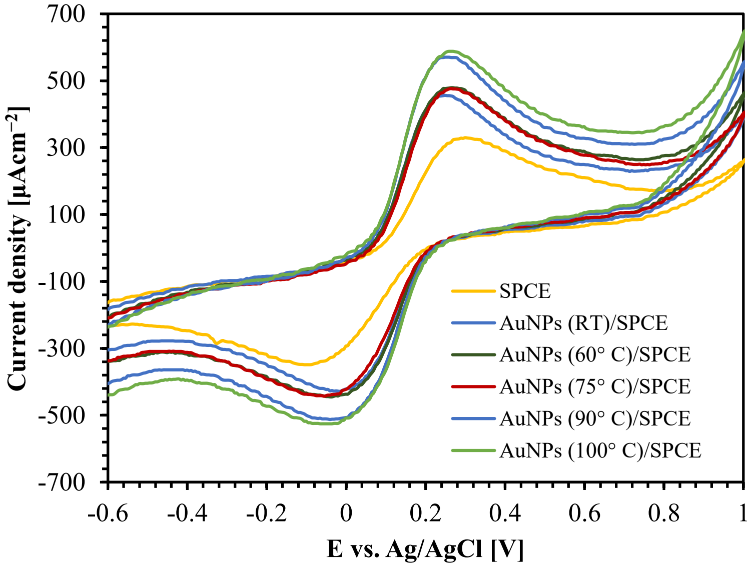

Fig. S6. CV responses of different AuNPs electrode in 5 mM $K_4[Fe(CN)_6]$ with 100 mM KCl at scan rate 60 mV/s. AuNPs were synthesised at different temperature (temperature (RT= 20± 2° C, 60° C, 75° C, 90° C and 100° C) using 0.5 mM $HAuCl_4$ and orange peel extract.

Effect of concentration of NaOH

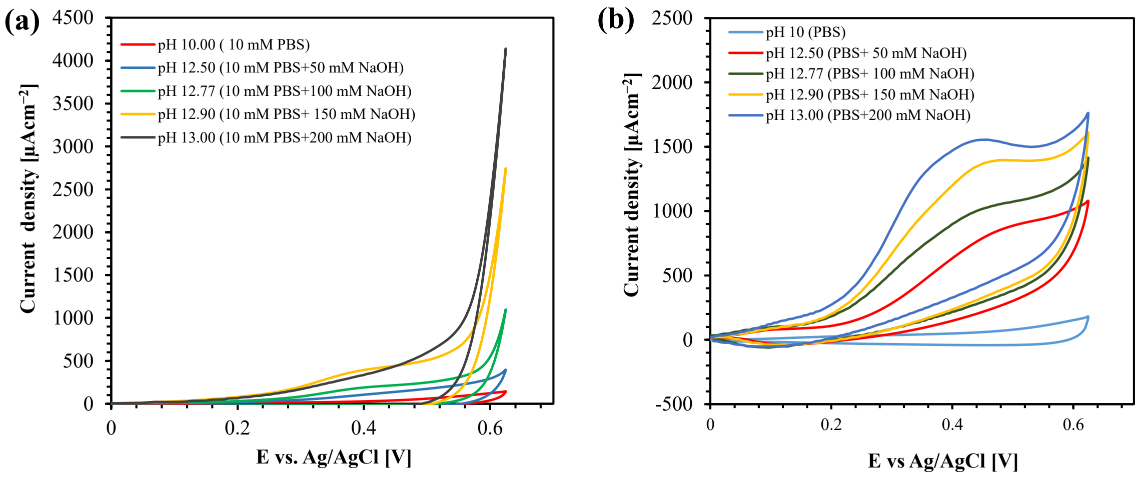

Fig. S7. Effect of pH and NaOH concentration (a) in the absence of glucose and (b) in the presence of 1 mM glucose at a scan rate of 60 mV/s

Effect of scan rate

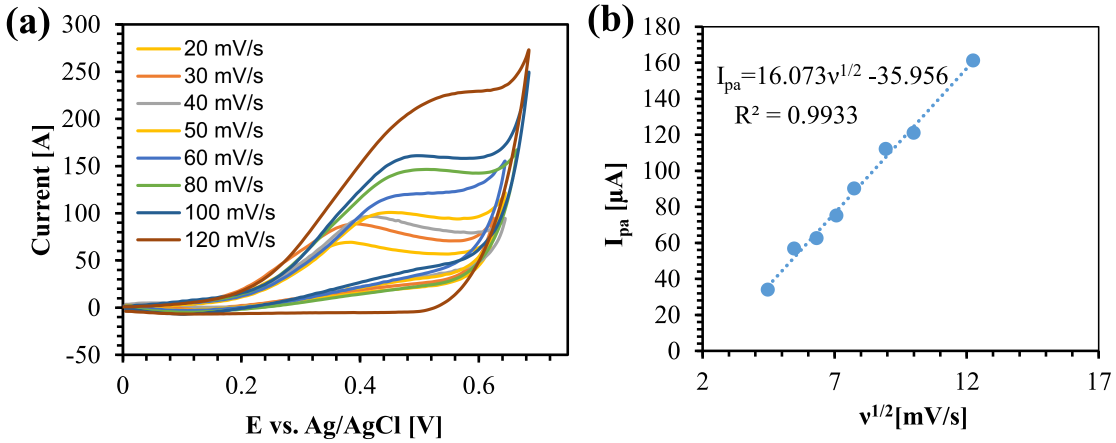

Fig. S8. (a) CV response of Au/CuO/SPCE in the presence of 1 mM glucose and 100 mM NaOH at different scan rate, (b) relationship between square root of scan (v) and peak oxidation current I_{pa}.

Reproducibility and stability of the sensor

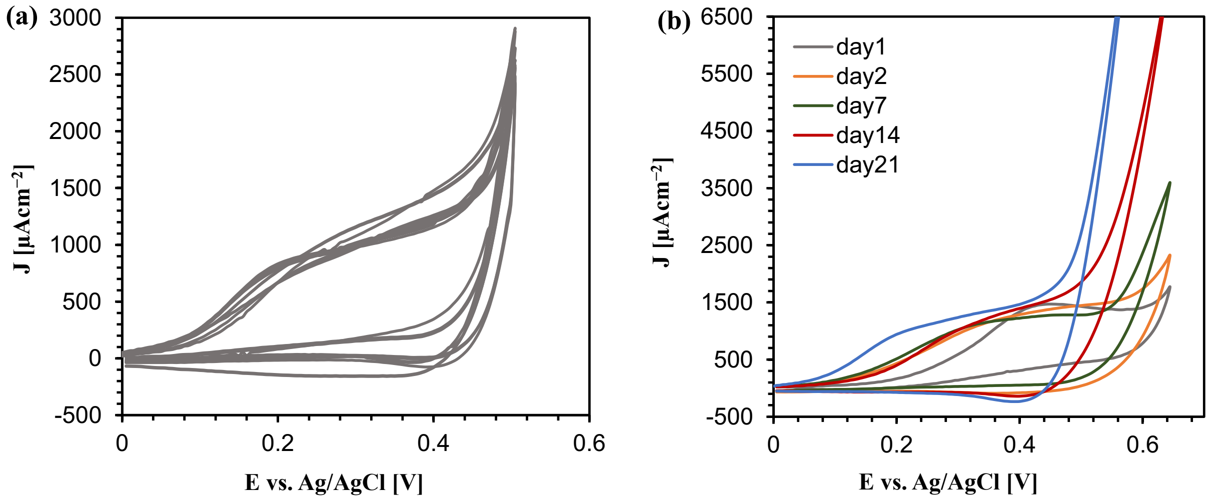

Fig. S9. (a) CV response of 10 Au/CuO/SPCEs on the same day after the sinthesis (b) CV response of Au/CuO/SPCEs electrodes at different days in the presence of 1 mM glucose in 0.1NaOH at scan rate 60 mV/s.
